# Co-designing an Adaption of a Mobile App to Enhance Communication, Safety, and Well-being Among People Living at Home With Early-Stage Dementia: Protocol for an Exploratory Multiple Case Study

**DOI:** 10.2196/19543

**Published:** 2021-12-20

**Authors:** Karen Davies, Sudeh Cheraghi-Sohi, Bie Nio Ong, Sudeh Cheraghi-Sohi, Katherine Perryman, Caroline Sanders

**Affiliations:** 1 National Institute for Health Research Patient Safety Research Translation Centre University of Manchester Manchester United Kingdom; 2 Primary Care Department Keele University Keele United Kingdom; 3 National Institute for Heath Resaerch School for Primary Care Research Keele United Kingdom

**Keywords:** design research, co-design, dementia, mobile app, communication, safety, mobile phone

## Abstract

**Background:**

There is a growing interest in using mobile apps to support communication, safety, and well-being. Evidence directly from people with dementia regarding the usability, usefulness, and relevance of mobile apps is limited.

**Objective:**

This paper describes the protocol of a study that will evaluate an app designed for supporting communication, safety, and well-being among people living with dementia. The study aims to understand if the app can enhance safety through improved communication among users.

**Methods:**

The study will use participatory qualitative methods over 3 cycles of evaluation with co-designers (service users, their families, and care practitioners). The study will be developed in partnership with a specialist home care service in England. Purposive case selection will be performed to ensure that the cases exemplify differences in experiences. The app will be evaluated in a walk-through workshop by people living with early-stage dementia and then trialed at home by up to 12 families in a try-out cycle. An amended version will be evaluated in a final walk-through workshop during cycle 3. Data will be collected from at least 4 data sources during the try-out phase and analyzed thematically. An explanatory multiple case study design will be used to synthesize and present the evidence from the three cycles, drawing on the Normalization Process Theory to support the interpretation of the findings.

**Results:**

The study is ready to be implemented, but it was paused to protect vulnerable individuals during the COVID-19 pandemic in 2020. The findings will be particularly relevant for understanding how to support vulnerable people living in the community during social distancing and the period following the pandemic as well as for providing insight into the challenges of social isolation that arise from living with dementia.

**Conclusions:**

Evaluating a mobile app for enhancing communication, safety, and well-being among people living with dementia contributes to the key ambitions enshrined in policy and practice—championing the use of digital technology and supporting people with dementia to live safely in their own homes. The study will involve co-designers living with dementia, so that the voices of service users can be used to highlight the benefits and challenges of assistive technology and shape the future development of apps that enhance safety by improving communication.

**International Registered Report Identifier (IRRID):**

PRR1-10.2196/19543

## Introduction

### Background

This paper presents the protocol of a study that will examine an app that is used and managed by people living with dementia and their families to improve communication and thereby reduce risks and enhance safety. Many people living with dementia have complex health and well-being needs that require carefully balanced support plans that need to be implemented across multiple settings where care takes place [1]. Complicated care regimes involving a range of people, together with variable or deteriorating cognitive abilities that affect communication, can pose significant risks for people living with dementia. Confusion can be compounded by complex health and social care systems that are difficult to navigate [2,3], and this can add to potential risks to safety. Minimizing the risks associated with the poor management of health among people living with dementia is a priority for governments [4] and family carers [5]. The rapid development of digital technology has enhanced timely communication in society and offers innovative ways to support communication, safety, and well-being for older people [6,7]. Previous studies that investigated the role of digital technology in supporting safety for people living with dementia focused mainly on passive monitoring for people with late-stage dementia [8]. Fewer examples of interventions for people living with dementia take account of the outcomes that they and their families identify as priorities [5]. In research that has gathered user views of safety in primary care, people have identified the importance of basic physical needs, such as being clean, comfortable, safe, and secure at home, but have also emphasized social and emotional concerns, such as feeling respected and being able to see, hear, and understand in order to communicate with others [9].

### Safety for People Living With Dementia

**Figure 1:**
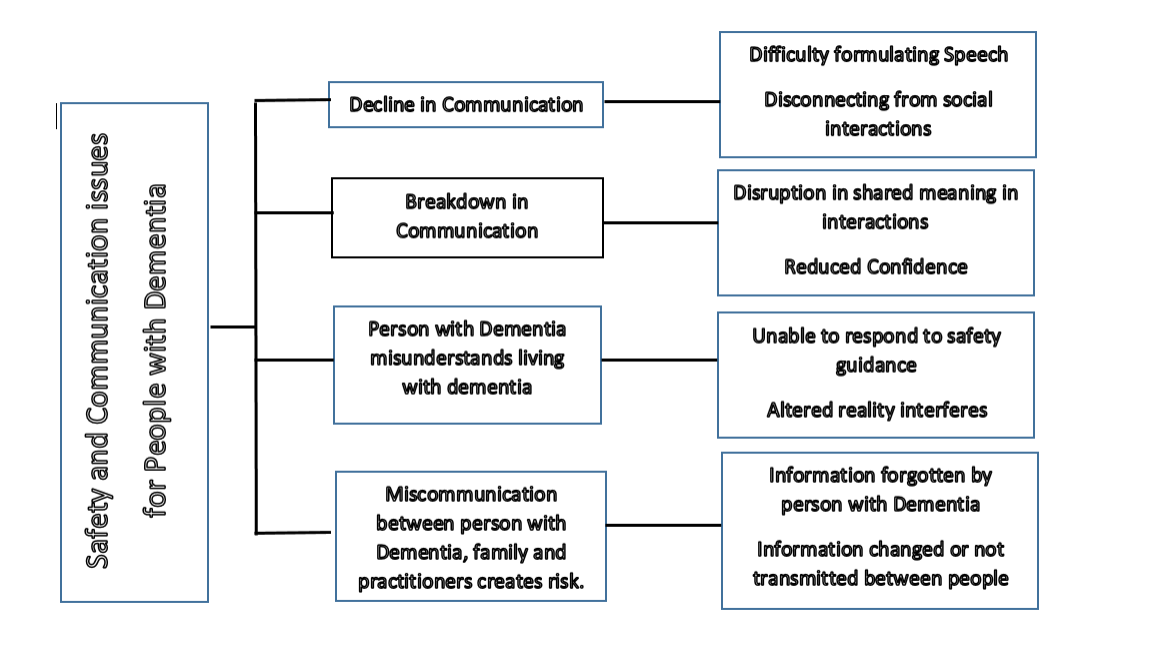
The relationship between communication issues for people living with dementia and safety.

Dementia is a neurological condition that is typified by a decline in brain function and structure and affects cognitive processing, such as memory, communication, and the control of physical movement [10]. There are different forms of dementia, and these are characterized by specific symptoms and the progression of the disease. However, the characteristics of progressive confusion, difficulty with managing routine activities, and limited communication are always evident and are often associated with the reduced awareness of risk and increased tendency to have accidents. For example, in 2012 to 2013, 18% of short-stay emergency admissions to hospitals among people living with dementia in England were related to injuries [11]. A number of safety issues have been reported in the literature [12], including injuries (eg, those related to falls, the ingestion of dangerous substances, sharp objects, fires and burns, etc), behaviors (eg, the act of wandering and getting lost, the inability to respond rapidly to a crisis, aggressive behavior, etc), and the misuse of medication [13]. The progressive nature of dementia, together with the process of distancing oneself from social interaction (which is linked to limited cognition and communication), may deepen an individual’s vulnerability and susceptibility to compromised safety, as illustrated in Figure 1.

The principal management of the condition involves alleviating the symptoms in order to maintain an active life, so that people living with dementia can frequently continue to live independently and safely with support from family and friends in their own homes [14]. Thus, there has been a shift in emphasis from finding a cure for dementia to seeking life-enhancing strategies for supporting people with dementia from within their homes (ie, strategies that are based on the unique contexts of their lives). Such an approach emphasizes relationships and interactions with family and the wider community, as embodied in the concept of personhood [15,16] and relationship-centered care [17]. Service users value positive and trusting relationships with care professionals and effective communication when they consider patient safety in primary care [9] and endorse the importance of enhancing these factors for people living with dementia.

### Implications of Communication Difficulties for Safety Specific to People Living With Dementia

Relationship-centered care depends on effective interaction and communication between people living with dementia and those in their social network; this includes interactions among professionals, formal carers, family, and friends [18]. The cognitive deficits that are present in people living with dementia will always be associated with declining language and communication skills, with distinctive difficulties and strengths being evident in the early, middle, and late stages of dementia (Textbox 1). Early-stage dementia [19]—a term that is frequently used to refer to people who are at the beginning stage of cognitive impairment, irrespective of the type of diagnosis—is characterized by mild problems in communicating as well as forgetfulness. These occur in the context of continuing independence in daily living [20]. Specific patterns of communication difficulties that are linked to types of dementia have been identified [21,22], but 3 general features need to be considered when developing an intervention to improve communication. First, communication is a social act that depends on interaction and conversation rather than on a simple transaction of information (ie, receiving information from and providing information to people living with dementia) [23]. Communication breakdown results from the interplay between two individuals—an individual with declining language skills and their conversation partner—suggesting that an intervention must enhance the interactions between people living with dementia and others in their social world. This applies to both verbal and written communication. Second, changes in sensory skills, such as visual and auditory acuity, affect the way that communication is scaffolded [23]; it would be a mistake to assume that audio and written words, for example, automatically augment communication. Third, people living with dementia undergo changes in how they experience reality, such as increasing confusion and disorientation as the condition progresses, that affect how they interpret the world. This also affects their ability to understand what they themselves, as well as people in their social network, say. Enhancing communication to support patient safety has been identified as 1 of the top 10 priorities by care practitioners and service users [24]. However, there is little empirical or theoretically backed evidence underpinning the best approaches to designing an intervention that supports communication with people living with dementia [23,25] and enhances safety [26].

**Characteristics of communication difficulties and strengths in dementia [23].**

**Communication and cognitive difficulties**

- Early-stage dementia
  Mild difficulty with remembering names and places
  Difficulty with abstract language and conversation
  Mild difficulty with memory and visuospatial activities
  Occasional lapses in attention
- Middle-stage dementia
  Increasing difficulty with word finding and the reduced use of “content” words
  Difficulty with understanding complex instructions
  Increasing difficulty with memory, attention, and the maintaining of a topic of conversation
  Difficulty with organizing and planning
- Late-stage dementia
  Significant difficulty with expressing needs
  Inappropriate verbal and vocal productions
  Lack of any speech in some cases
  Severe difficulty with understanding spoken language
  Severe memory difficulties
  Difficulty with maintaining attention

**Communication and cognitive strengths**

- Early-stage dementia
  Talks in full sentences and maintains conversation appropriately
  Maintains an understanding of concrete language
  Aware of difficulties
- Middle-stage dementia
  Conversational turn-taking is maintained
  Able to read aloud and understand familiar written phrases
  Maintains familiar, overlearned skills such as brushing hair and drinking from a cup
- Late-stage dementia
  Appropriate affective responses to sensory stimuli and music
  Able to cooperate with appropriate cues (touch, vision, and emotion)

### Dementia and Assistive Technology

Assistive technology has become a central element of policy and guidance for improving the care of people living with dementia [4,27,28], although issues of adoption by people living with dementia remain challenging [29]. Assistive technology can operate as an aid for maintaining or improving a person’s functioning or independence [30]. The Alzheimer’s Society [31] suggests that these technologies can serve the following three roles: (1) supportive technologies that enable people to complete tasks, (2) responsive technologies that manage risk and raise alarms, and (3) preventative technologies that prevent harm and raise alarms. Assistive technologies can take the form of simple low-tech equipment, such as walking aids, or high-tech aids that make use of digital technology to offer support.

The largest growth in the use of assistive technology for older people has focused on safety and security in the community [29]. However, these technologies often come in the form of passive devices, such smoke detectors, that do not require participation by people living with dementia. Studies investigating assistive technologies that require active involvement and engagement with devices have indicated that people living with dementia do not use the safety-related features of assistive technology devices. Even when a digital assistive device has been codeveloped with people living with dementia, there appears to be a disparity between acceptance and use [32,33]. A recent scoping review reported on the importance that older adults place on expressing their identity as one of independence, self-reliance, and competence, which influences their decisions about adopting technological solutions [34]. The cultural meaning attached to digital devices is evident in society; one item (eg, a smartphone) can symbolize independence and modernity, while another (eg, alarms) may symbolize stigma and dependence [34]. Using devices may be perceived by some people as an action that reinforces negative identities and ageist stereotypes [35].

Smart mobile and wearable technology, in the form of apps, can help overcome any issues of user acceptability resulting from assistive technology being “concealed” in everyday technology such as smartphones. Such integrated technology therefore allows for the real-time monitoring of health variables and social aspects of an older person’s life [36]. Taken together, these characteristics offer promising outcomes. However, the proliferation of apps for health care has not been matched by robust research, and major criticisms have been aimed at the limited evaluation of clinical outcomes [30,37]. Principles for developing and applying app technology are emerging [30], with personalization being expressed as a priority [30,38]. Other essential considerations include the quality of the content, usability, the matching of apps to users’ health literacy levels, and security and privacy issues [39].

### Hear Me Now App

In England, the National Health Service has created a library of apps and web-based tools that aim to help people manage their health and well-being [40]. They have been approved in terms of clinical safety, data protection, security, and usability. However, at the time of writing this paper, there are only 2 apps in this library that are specific to dementia. Although a general web search indicates a proliferation of apps, few have been evaluated [41]. The Hear Me Now (HMN) app (formerly the My Health Guide app) [42] was developed to help people with learning disabilities and those who support them be in control of their health and well-being information. People can use the app to explain their needs and concerns, help them understand how to act on advice given by health and social care professionals, keep and share information about themselves and their needs, and record consultations to listen to them again at a later date. Furthermore, HMN supports care practitioners in understanding an individual’s needs better by providing quick access to key information that facilitates communication and interaction with app users.

### Normalization Process Theory

HMN depends on interacting components, such as users’ understanding; the usability of the app; and the app’s adoption, which is contingent on individual, group, and organizational behaviors. It can be considered to be a complex intervention [43]. The Normalization Process Theory (NPT) was developed to improve people’s understanding of how complex interventions work. The theory looks beyond the workability of systems [44] and instead looks at issues such as the effects that eHealth interventions have on the roles and responsibilities of all those involved, how interventions affect interactions between service users and care practitioners and the outcomes of service users, and implications for care practitioners. The NPT is a middle-range theory that explores issues of how interventions work in practice in more depth. It provides a framework for conducting multifaceted analyses to understand the actions and interactions influencing implementation and how new interventions and practices come to be normalized in health and social care contexts. Conversely, it can aid with the explanation of why technologies fail to be routinely adopted when they are implemented in organizational contexts [45]. There are 4 key constructs that can be applied to service users and care practitioners who use digital apps. First, the construct of *coherence* is used to describe the way individuals understand the meaning of a new technology and its associated practices. Second, the construct of *cognitive participation* explains the relational work that is needed to sustain a community of practice for a new intervention. Third, the *collective action* construct signifies the operational work required to enact new practices. Fourth, the *reflexive monitoring* construct applies to the appraisal of new practices. The NPT examines the processes of changes that occur in individual and collective behaviors and allows the dynamics of human agency to be connected to a given context. The theory has explanatory power; thus, it can be used to understand the work that is required for the adoption and integration of new interventions.

The aims of our study are to (1) evaluate the usability, usefulness, and relevance of HMN among people living with early-stage dementia who live in the community; (2) examine the benefits and challenges of using HMN to enhance the management of health, safety, and well-being; and (3) determine if adaptions are required in HMN and how these adaptations should be designed.

## Methods

### Study Design

**Figure 2:**
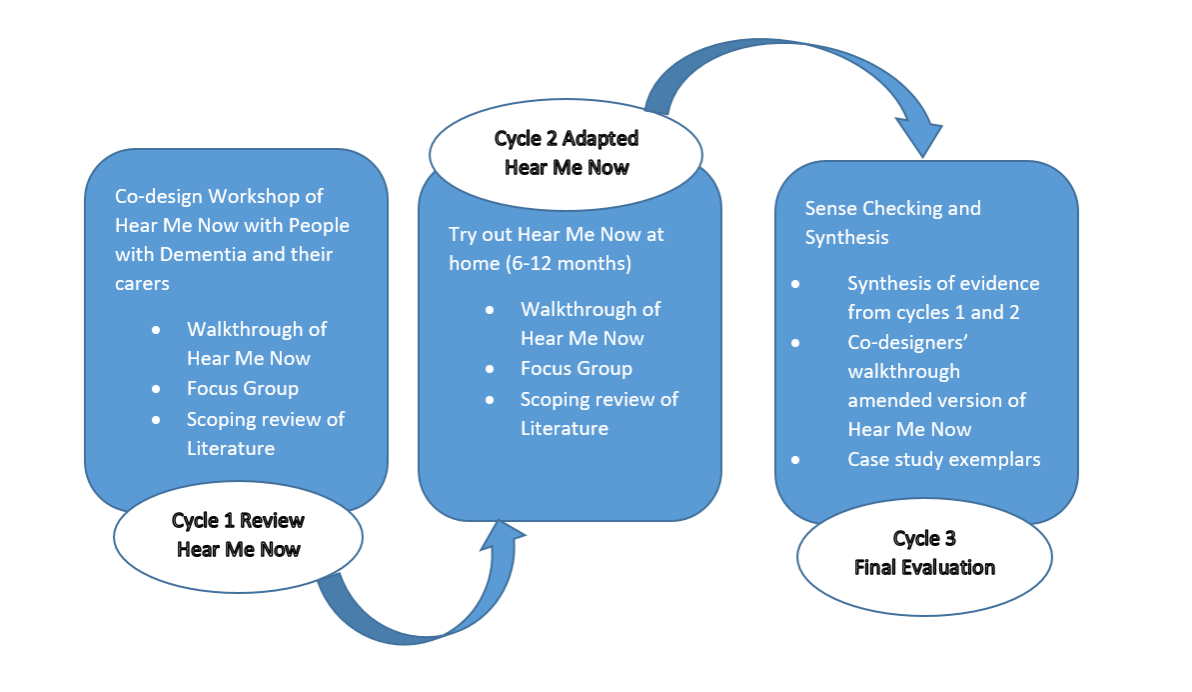
Cycles of Design research Hear Me Now.

We will conduct a formative evaluation based on the principles of design research [46,47] by involving co-designers (service users, their families, and care practitioners) in 3 evaluation cycles, in which data will be collected over 18 months (Figure 2). There will be a 6-month preparation phase, during which patient and public involvement and engagement strategies will be used before the evaluation commences. Co-design studies involve designers and nondesigners working together to design, or potentially redesign, a product. In this study, the digital design company Maldaba, which is experienced in designing HMN for people with a learning disability, will be working with people living with dementia, care practitioners, and researchers to redesign the app [42]. The previous evaluation of the app showed that HMN was used for creating appointment reminders, documenting information about an individual, recording medical information, and noting people’s feelings. The results suggested that the app was easy to operate, but the availability of support was an important factor in the adoption and use of the app. Furthermore, users had a preference for using the app to interact and converse with individuals in their informal support networks [48].

Multiple individual case studies will be used to record and report in-depth evidence of the app’s usability, usefulness, and relevance among people living with dementia, and a number of sources of data (eg, interviews, demographics, analytics, etc) will be used to help us understand an individual participant’s case. Yin’s [49] 6-stage case study process will be used, together with other guidance [50], to support the rigor of the design. Case studies are valuable for exploratory and explanatory purposes and are suited to contemporary phenomena for which “how” and “why” questions are posed. They are particularly appropriate for research questions that are posed to understand and evaluate the complexities of implementing and adapting an existing intervention with a new group of service users. Purposive case selection will be conducted to ensure that the cases illustrate a variety of contexts. Data will be analyzed by using deductive and inductive approaches.

The research will be conducted over 24 months and involve the following steps:

- Preparation (months 1-6): develop an understanding of issues and processes with patient and public involvement and engagement strategies, negotiate arrangements with partners, obtain ethical approval, set up an advisory group, and prepare walk-through workshop
- Cycle 1 (months 6-12): the collection of data for review and the remodeling of HMN in a walk-through workshop with up to 24 co-designers (people living with dementia, carers, and care practitioners)
- Cycle 2 (months 12-18): recruiting and working with up to 12 co-designers with dementia and up to 12 people from their health and social networks
- Cycle 3 (month 18): final walk-through workshop and sense-checking event with co-designers (end-of-project workshop)
- Reporting (months 18-24): written report, dissemination with co-designers, impact activities

### The Intervention: HMN

HMN is a user-led app that collates users’ preferences so that users can engage with the app in a relevant and personalized way (ie, according to their own needs and experiences). App users (and their families) are able to build a customized profile containing relevant information that they can control, such as information on how best to communicate with others, personal interests and concerns, and essential information. Information can be captured and presented in different formats, such as audio, text, and visuals, and written content can be verbalized to users via the app simply by clicking the text-to-speech icon. The key features (Textbox 2) of the intervention were identified during development and evaluation with people with a learning disability [48].

**Key features of Hear Me Now.**

**App users control the content**

- Users have control over how the app is used by creating boxes to store things that are important to them. Boxes can be labeled by using photos or text.

**Enables flexible use**

- Each box can store content that is captured as text, pictures, videos, or audio.
- Content can be created by using the app, and users can add content that is already stored on their device.
- Written content can be verbalized to users by clicking on a text-to-speech icon

**Enables personalization**

- The app prioritizes “important things about me,” thereby enabling users to build a life story that can be shared with other people when they attend appointments or social events.
- App users can personalize the app. This includes adding a profile photo, changing the color and font size of text, creating reminders, and using an in-app personal ID number for security.

**Contains an appointment facility**

- The app includes an appointment facility for creating appointments.

**Contains contact information and a sharing facility**

- Users can record the details of friends, family, and carers and can share their boxes with anyone in their contact list. Contacts are notified via email and can log into the Hear Me Now web interface on their browser. The web interface lets contacts do the following:
  Stay in touch with the app user’s latest activities
  Suggest content additions for the boxes they can see (app users will be able to accept or decline additions)
  Send app users alerts (the app user will see the alert next time their app synchronizes with the server)

**Able to store documents**

- Documents can be uploaded to the app and stored in 1 place

### Participants

Participants will be recruited, together with partners who deliver home care, from the northwest of England over 12 months based on the inclusion and exclusion criteria outlined in Textbox 3. Participants with early-stage dementia (as identified by people living with dementia and their carers) who live at home will be introduced to the study by home care practitioners, who routinely visit people with dementia living at home. They will provide study information leaflets and introduce the researchers to the families of people living with dementia who express an interest in participating in the study. The sampling will be purposive in order to have participants with varied experiences of dementia, backgrounds, and living arrangements.

**Inclusion and exclusion criteria.**

**Inclusion criteria**

- Medical diagnosis of dementia
- People living with early-stage dementia
- Living in own home or extra care housing
- Experiences symptoms of memory loss and cognitive changes but lives independently
- Adequate vision and motor skills for managing a tablet computer
- Supported by family carer and/or formal carers
- General health is stable and well managed
- Able to converse in English

**Exclusion criteria**

- No diagnosis of dementia
- People living with late-stage dementia
- Living in a care home
- Extensive cognitive confusion
- Unable to handle a tablet computer
- Receiving no support from carers
- Experiencing regular bouts of acute illness
- Limited spoken English

As the data collected from each participant will be predominantly qualitative (data will be drawn from a number sources for detailed case studies), a total of 12 participants with dementia will be recruited, together with family members who offer support (spouses, partners, and children), formal carers, and regular care practitioners (up to 24 in total). Formal carers and care practitioners will be those who have routine involvement with the person with dementia (at least 1 contact per week).

### Data Sources and Data Collection for Case-Based Evidence

Design research is conducted iteratively in collaboration with co-designers. Formative evaluations provide the opportunity to improve the design of an intervention and are integral to the design process. Three iterative cycles of review, formative evaluation, and redesign will be conducted (Figure 2) by using a range of data sources to triangulate the evidence. In the first cycle, a workshop for a walk-through of the current app will be conducted in the community with people with early-stage dementia, their carers, and care practitioners to review the current version of HMN. This will involve investigating the usability, usefulness, and relevance of the app. The walk-through method is a part of the user experience design process that is used in software development. It involves a step-by-step process in which technology users, as they deploy an app, observe practicality, note users’ comments, and seek a detailed narrative of use [51].

The walk-through will be conducted remotely (where possible) with the assistance of carers and support staff. It will involve qualitative data collection methods, including the observation of app use, interviews for gathering data on individuals’ experiences with using the app, and focus groups conducted via videoconferencing for examining the collective views of service users. Each set of data will be analyzed thematically. Potential changes and recommendations for the redesign will be discussed and enacted by the app developers [42].

The second cycle will involve 12 people with dementia who live independently and associated family members and care practitioners using the app during everyday life. Each case will be contacted (2 contacts over 3-6 months) to collect a range of data as participants use the app within their homes. This will be done remotely or by care practitioners visiting families (Textbox 4). The third cycle will involve the analysis of the data from cycle 2 together with the data generated at the final walk-through and sense-checking workshop, which will include people living with dementia, their families, and care practitioners.

**Summary of data sources and data collection details for cycle 2.**

**Usage logs**

- Usage data (the frequency of use and length of time using the app) will be collected from all participants during the data collection period (in collaboration with the developers).

**Semistructured interviews**

- One-to-one semistructured interviews with people living with dementia, people in their social networks, and professionals will be conducted at 2 time points to assess the initial and ongoing use of and experiences with the app over a time frame of up to 12 months. These interviews will be adapted appropriately to the cognitive status of individuals with dementia and undertaken by using videoconferencing software or phones to ensure participants’ safety during the pandemic. Researchers will undertake appropriate training to conduct interviews with people living with dementia. Interviews will be recorded and uploaded to NVivo (QSR International) for automated transcription.
- The interviews will be conducted by using a topic guide, which will be based on the objectives of the Hear Me Now app, along with questions for exploring the potential of the app to facilitate communication with health and social care professionals. The topic guide will be used flexibly in interviews, and emerging themes will be explored in subsequent interviews in accordance with the conventions of qualitative data collection.

**Observation of app use**

- In order to obtain contextual data on people using the app in different contexts, we aim to observe up to 12 people living with dementia (with or without key people in their social networks) in each setting (homes and extra care housing). We will observe participants using the app via videos of a prearranged visit when people living with dementia receive an episode of care. Observations will focus on the interactions between people living with dementia and a regular formal carer or professional, such as the admiral nurse. These will be carried out by conducting remote data collection and analyzed in a structured format. The analysis will be augmented by using field notes from the initial observations of video interactions.

**Review of documents uploaded to app**

- Documentary data will be collected in the form of photographs, drawings, and other visual data. Participants will decide whether they want to produce these materials for research purposes, and if so, they will choose the formats that are the most relevant to them. More formal documents, such as relevant notes of meetings (case conferences) or action plans, will be collected. Documentary data will be summarized by using structured forms that are based on the key features of Hear Me Now.

### Data Analysis

The qualitative data analysis will be conducted via an abductive approach [52], thereby enabling a continuous dialogue among theory, data from users’ experiences, and existing models of engagement. This approach allows for the conduction of inductive and deductive analyses, which ensure that findings are based on both new empirical evidence and previous evidence. This will align well with the iterative design process and the cycles of evidence collection and redesign for the HMN app. The analysis will be conducted by two researchers who are trained and experienced in qualitative research. Interviews and focus groups will be audio recorded, transcribed, and analyzed thematically to identify key themes [53]. Participants’ use of the app during interactions with family and care practitioners in their usual settings will be recorded via direct, nonparticipant observation by using a structured template and field notes. These will be also be analyzed thematically [54]. NVivo (QSR International) software will be used to support the management and analysis of data. In keeping with qualitative research conventions, the involvement of the user participants will provide the opportunity to conduct sensemaking exercises to assure the participants of the trustworthiness and credibility of the data analysis and interpretation. The app developers will form part of the research team; they will provide their expertise to support the interpretation of findings, identify areas for redesign, and prepare amended prototypes for evaluation during the cycles of the design research process. A framework analysis will be conducted to collate and analyze the data for the case studies in order to describe, explain, and compare the similarities and differences among cases. The comparison with NPT constructs should provide further depth to the conceptual analysis, and this will contribute to the final evaluation of the HMN app for people living with dementia.

### Patient and Public Involvement and Engagement

The study will use participatory methods for involving patients and the public in the study as part of the design process. For the purposes of maintaining governance and ensuring the involvement of professionals in the co-design (in keeping with the 2018 guidance from the National Institute for Health and Care Excellence) [55], we will establish a “critical friends” group for guidance in designing the study. This will consist of a person living with dementia, a care practitioner, and an academic experienced in dementia research. The trustworthiness and credibility of the findings will be enhanced through the involvement of external partners in peer-review and sense-checking research plans, the dissemination of findings, and data interpretation. Specific tasks will include commenting on the study design and study documents, developing and conducting the data collection and analysis processes, and disseminating the findings.

### Ethics and Governance

We are following ethical guidance from the University of Manchester ethics committee and have obtained approval in accordance with their standardized system. The recruitment, data transfer, and data storage processes are in accordance with the legislation and guidance from the research institute. Written informed consent will be obtained from all participants, and arrangements are in place for anonymizing and securely storing obtained data. The study participants will be at the early stage of dementia and will have the capacity to consent. If the participants’ capacity to consent changes during the research cycles, researchers will adopt the Mental Capacity Act Code of Practice and work alongside experienced care practitioners to ensure that appropriate checks are in place. The study will be managed on a day-to-day basis by a project management group consisting of experienced researchers who will meet monthly to make plans and monitor progress. A “critical friends” group will be established to provide guidance for the development of study materials, conduction of analyses, and interpretation and dissemination of findings.

## Results

Digital technology is developing rapidly, and using apps to manage the many elements of daily life is becoming routine. Given the context of social distancing, which was introduced to combat the COVID-19 pandemic in 2020, it is particularly urgent to understand the views of people living with dementia and those in their social and care networks regarding the use of an app that focuses on supporting communication, safety, and well-being. Our study will enable us to evaluate HMN’s usability and its value for people living with dementia and identify adaptions for improving the app’s implementation. The study will consider how using the app might contribute to communication and potentially contribute to improving safety through a personalized approach to recording health and well-being issues that are important to people living with dementia in the community. The study will also contribute to methodological innovations, as it will involve illustrative, NPT-informed case studies of how an app is used by people at home. This will provide the basis for an explanatory model of the relationship among factors such as communication, social and health care networks, cognitive strengths and weaknesses, and the enhancement of safety in the community.

Ethics approval was obtained from the University of Manchester Ethics Committee (reference number: 2020-8665-13751). Participants will give written consent to participate in the study and consent to the publication of anonymized data. Supporting data will be made available upon request from the corresponding author.

## Discussion

Evaluating a mobile app for enhancing communication, safety, and well-being among people living with dementia contributes to the key ambitions enshrined in policy and practice—championing the use of digital technology and supporting people with dementia to live safely in their own homes. The study will involve co-designers living with dementia, so that the voices of service users can be used to highlight the benefits and challenges of assistive technology and shape the future development of apps that enhance safety by improving communication.

## Abbreviations

HMN: Hear Me Now
NPT: Normalization Process Theory
